# Unveiling the Tumor Microenvironment Through Fibroblast Activation Protein Targeting in Diagnostic Nuclear Medicine: A Didactic Review on Biological Rationales and Key Imaging Agents

**DOI:** 10.3390/biology13120967

**Published:** 2024-11-24

**Authors:** Juliette Fouillet, Jade Torchio, Léa Rubira, Cyril Fersing

**Affiliations:** 1 Nuclear Medicine Department, Institut Régional du Cancer de Montpellier (ICM), University Montpellier, 34090 Montpellier, France; 2 IBMM, University Montpellier, CNRS, ENSCM, 34293 Montpellier, France

**Keywords:** tumor microenvironment, cancer associated fibroblasts, fibroblast activation protein, nuclear medicine, molecular imaging, radiopharmaceuticals

## Abstract

The tumor microenvironment (TME) is a dynamic and complex medium that plays a central role in cancer progression, metastasis, and treatment resistance. Among the key elements of the TME, cancer-associated fibroblasts (CAFs) are particularly important for their ability to remodel the extracellular matrix, promote angiogenesis, and suppress anti-tumor immune responses. Fibroblast activation protein (FAP), predominantly expressed by CAFs, has emerged as a promising target in both cancer diagnostics and therapeutics. In nuclear medicine, targeting FAP offers new opportunities for non-invasive imaging using radiolabeled fibroblast activation protein inhibitors (FAPIs). These FAP-specific radiotracers have demonstrated excellent tumor detection properties compared to traditional radiopharmaceuticals such as [^18^F]FDG, especially in cancers with low metabolic activity, like liver and biliary tract tumors. The most recent FAPI derivatives not only enhance the accuracy of positron emission tomography (PET) imaging but also hold potential for theranostic applications by delivering targeted radionuclide therapies. This review examines the biological underpinnings of FAP in the TME, the design of FAPI-based imaging agents, and their evolving role in cancer diagnostics, highlighting the potential of FAP as a target for precision oncology.

## Simple Summary

The environment of cancer cells, made up of multiple cell types, macromolecules, and signaling molecules, displays numerous properties that promote cancerous diseases. This tumor microenvironment is characterized by a high degree of diversity, offering a substantial number of specific markers that can be exploited to target tumor processes, both for diagnosis and therapy. The fibroblast activation protein, overexpressed on the surface of cancer-associated fibroblasts, has attracted considerable interest through the design of inhibitors, used as radiolabeled molecular imaging probes in nuclear medicine. Some of these experimental radiopharmaceuticals have already been extensively studied in clinical settings, notably for cancer imaging when other molecular imaging techniques present limitations. Despite this, innovative analogs continue to be developed, some of which for both diagnostic and therapeutic applications.

## 1. Introduction

The tumor microenvironment (TME) plays a crucial role in cancer progression, significantly influencing how tumors grow, evade the immune system, and develop resistance to treatments [1]. Unlike early cancer models that primarily focused on tumor cells themselves, contemporary oncology places great emphasis on the TME as a complex ecosystem composed of stromal cells, immune cells, blood vessels, and extracellular matrix components [2]. This dynamic environment not only supports tumor cell survival and proliferation but also contributes to invasion and metastasis. Understanding the TME has therefore become a central concern in the development of more effective diagnostic and therapeutic strategies.

Among the various components of the TME, cancer-associated fibroblasts (CAFs) have gained increasing attention [3]. These stromal cells, particularly through their expression of fibroblast activation protein (FAP), are involved in cancer progression by modifying the extracellular matrix, promoting angiogenesis, and suppressing immune responses. FAP is highly expressed in CAFs within the TME, particularly in many epithelial tumors, making it an attractive target for diagnostic imaging and therapeutic approaches [4]. In this context, the development of FAP-targeted imaging agents has opened new frontiers in nuclear medicine, providing tools to visualize and quantify the TME influence in cancer. These agents, particularly FAP inhibitors (FAPIs), allow for non-invasive imaging that can complement existing modalities like [^18^F]FDG PET, offering high specificity and sensitivity in certain cancer types, revealing tumors that might otherwise go undetected [5].

Within this scope, the present review aims to provide a comprehensive examination of the biological rationale for targeting FAP in cancer imaging and therapy. First, the influence of the tumor microenvironment in cancer progression will be outlined, paying special attention to the interaction between immune cells, CAFs, and the extracellular matrix. Then, the pivotal role of CAFs in the TME will be specifically explored, with a focus on FAP biochemical properties and its contributions to tumorigenesis, highlighting why this enzyme is a critical player in the TME and a promising target for nuclear medicine. From there, the design, development, and clinical application of key FAP-targeting imaging agents will be detailed, with a focus on quinoline-based FAPI molecules. We will discuss their performance in clinical settings, comparing their efficacy to other oncology radiotracers such as [^18^F]FDG. Finally, an overview of the most recent and innovative FAPI derivatives (especially non-quinoline compounds) will be given, highlighting their respective advantages and level of clinical development.

## 2. A Dive into Tumor Microenvironment

### 2.1. General Considerations About Tumor Microenvironment

In 2000, Hanahan and Weinberg proposed six hallmark characteristics of cancer that tumor cells acquire during their development: sustained proliferative signaling, evasion from tumor suppression, resistance to cell death, replicative immortality, induction of angiogenesis, and activation of invasion and metastasis [6]. In 2011, two additional traits were highlighted: reprogramming of energy metabolism and evasion of immune destruction [7]. These hallmarks provide an essential framework for understanding the underlying mechanisms of cancer development and progression.

**Figure 1:**
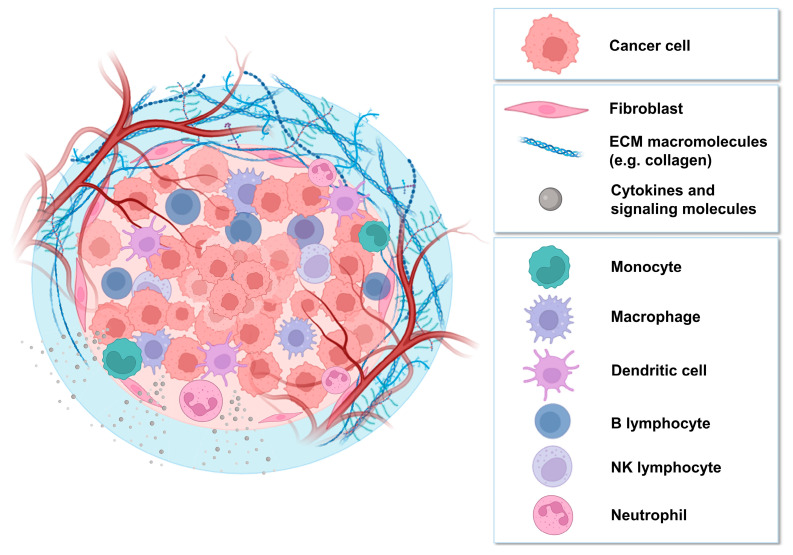
Summary of the main cell types and molecules found in the tumor microenvironment, grouped into 3 categories: cancer cells (first box), extracellular matrix (second box), and immune cells (third box).

A solid tumor is defined by an aggregate of cancer cells, surrounded by various cell types, as well as extracellular matrix (ECM) and signaling molecules, which together form the tumor microenvironment (TME) [8]. Although the composition of the TME is influenced by the tumor type, several elements are consistently found in the stroma, such as immune cells (monocytes, macrophages, dendritic cells, lymphocytes, and neutrophils); stromal cells (fibroblasts and endothelial cells); extracellular matrix components (glycoproteins, collagen, and enzymes); cytokines (IL-6, IL-17, and IL-23); and blood vessels [9]. Each of these components, illustrated in Figure 1, has immunological or mechanical functions that influence the tumor and TME, this heterogeneity creating a dynamic environment favorable to cancer cell growth, invasion, and survival [10].

### 2.2. The Roles of the Tumor Microenvironment

#### 2.2.1. Immune Interactions and Tumor Growth

**Figure 2:**
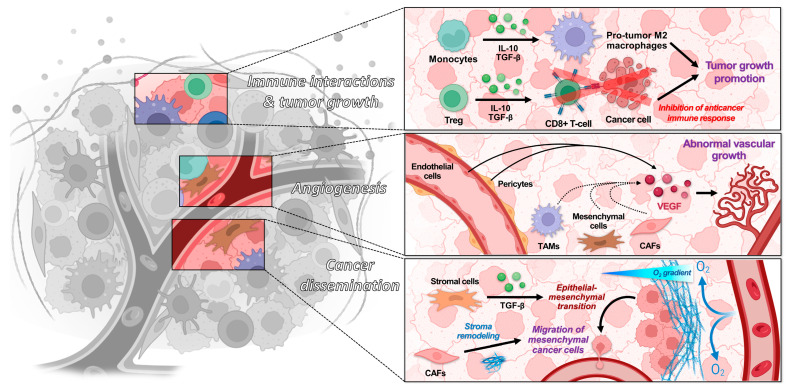
Simplified overview of the main tumor microenvironment functions, along with the major cell types and signaling molecules involved.

Within the TME, immune cells contribute to the activation of an inflammatory response that promotes the development and spread of tumor cells (Figure 2). Under certain circumstances, immune cells can also inhibit tumor progression [11]. It is important to note that the immune system operates through two distinct mechanisms: innate immunity and adaptive (or acquired) immunity. Adaptive immunity is a specific immune response developed in response to particular antigens, typically through exposure to pathogens or vaccines. T- and B-lymphocytes are the primary effectors of adaptive immunity. In contrast, innate immunity provides non-specific protection against a broad range of pathogens through mechanisms such as physical barriers, phagocytic cells, and antimicrobial proteins. Innate immunity involves monocytes, macrophages, dendritic cells, natural killer (NK) cells, and neutrophils (PNNs) [12]. Details on the effects of these different cell types are given in Section 2.3.

#### 2.2.2. Angiogenesis

Angiogenesis is a physiological process arising in cancer due to the tumor’s increasing demand for oxygen and nutrients. Tumor vascularization involves the cooperation of various cells within the TME, including vascular endothelial cells, pericytes, and bone marrow-derived precursor cells [13]. Other supporting cell types, such as tumor-associated macrophages (TAMs), mesenchymal cells, PNNs, and cancer-associated fibroblasts (CAFs), tend to enhance tumor vascularization by releasing pro-angiogenic signals within the TME (Figure 2) [14]. Similarly, lymphangiogenesis refers to the formation of new lymphatic vessels, which provide an alternative route for cancer cell dissemination. Activated macrophages produce growth factors that are correlated with lymphangiogenesis in certain cancers [15,16].

#### 2.2.3. Dissemination and Intercellular Interactions

Endothelial cells and stromal cells such as fibroblasts secrete growth factors that influence angiogenesis, tumor proliferation, and tumor invasion [17]. The epithelial–mesenchymal transition (EMT), during which tumor cells shift from an epithelial to a mesenchymal state, drives tumor dissemination, with a fluctuating phenotype depending on the environment [18]. The stromal cells plays a key role in these phenotypic transitions, particularly through the production or inhibition of transforming growth factor-beta (TGF-β) (Figure 2) [19]. Similarly, hepatic stellate cells are involved in the progression of hepatocellular carcinoma by remodeling the local ECM and creating a tumor-promoting environment [20,21,22].

The tumor margin represents the boundary between the tumor and healthy tissue. This region plays a critical role in the dynamic interactions between stromal and immune cells. Immature myeloid cells accumulate in this area, preventing the differentiation of dendritic cells into antigen-presenting cells, thereby facilitating immune evasion of the tumor. TAMs are recruited by tumor-derived chemotactic factors and promote tumor cell invasion by providing pro-migratory factors such as epithelial growth factor (EGF), regulating fibrillar collagen production to enhance tumor motility, and supporting proteolytic remodeling of the ECM [15,23]. CAF are abundant at the tumor margin and may release pro-invasive factors, further contributing to tumor progression [24]. Oxygenation levels at the tumor margin differ from the tumor core, with central hypoxia and better peripheral oxygenation. This hypoxia influences the behavior of stromal cells and their recruitment to the tumor; immune cells recruited by hypoxia concentrate at the periphery to support tumor invasion, and immune cells remaining at the tumor core promote the selection of resilient cancer cells, which subsequently migrate toward the tumor margin [13].

### 2.3. Tumor Microenvironment Components

#### 2.3.1. Innate Immune Cells

**Figure 3:**
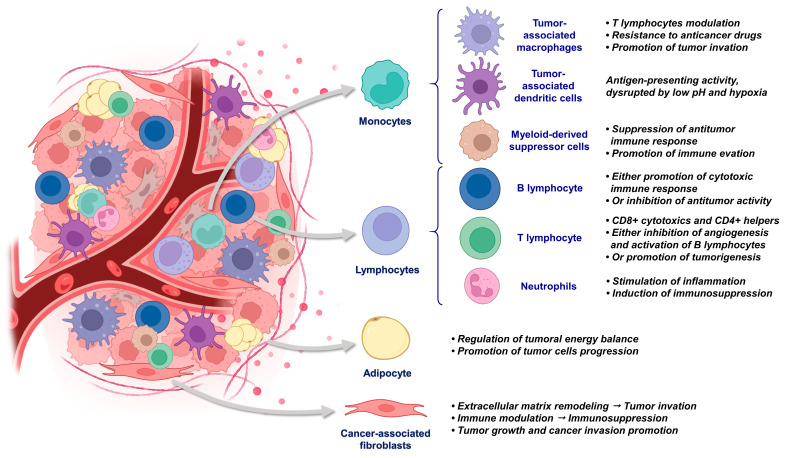
Summary of the essential properties of the main cell types found in the TME.

Monocytes originate from myelomonocytic stem cells and form a population of circulating cells capable of migrating into tissues in response to specific signals. Upon recruitment to the TME, monocytes undergo a differentiation process and differentiate into three distinct cell subtypes: TAMs, tumor-associated dendritic cells, and myeloid-derived suppressor cells (MDSCs) (Figure 3) [25].

The most commonly observed cell type in the TME is the macrophage [26]. These cells can induce resistance to various anti-tumor therapies, notably by modulating T-lymphocyte activity through immunoregulatory mechanisms such as programmed cell death protein-1 (PD-1) [26,27]. TAMs also promote invasive cellular phenotypes and produce proteases, facilitating tumor invasion [15,28]. TAMs can be polarized into either M1 phenotype (with anti-tumor activity) or M2 phenotype, driven by hypoxia and IL-4 (with pro-tumor activity) [10,29,30]. High TAM infiltration is often associated with poor prognosis in various cancers, including breast, lung, and gastric cancers [9].

Dendritic cells function as antigen-presenting cells, recognizing, capturing, and presenting antigens to T-lymphocytes in lymphoid organs. They bridge innate and adaptive immunity, but their function can be impaired within the TME [31]. Tumor dendritic cells exhibit deficiencies in activation and do not effectively stimulate the immune response, while conditions such as hypoxia and low pH further disrupt their function [32].

MDSCs represent a heterogeneous myeloid population that suppresses anti-tumor immune responses and promotes immune evasion [25]. These immature cells hinder antigen presentation, T cell activation, and NK cell function, thereby facilitating tumor progression. Elevated levels of MDSCs in cancer patients are usually associated with rapid disease progression and reduced therapeutic efficacy [30].

#### 2.3.2. Adaptive Immunity Cells

Like macrophages, lymphocytes are a key component of the TME [10]. Depending on their differentiation, lymphocytes can exert both pro- and anti-tumor effects. Two types of lymphocytes can be found in the TME: B-lymphocytes and T-lymphocytes (Figure 3).

B-lymphocytes, the main cellular elements of the humoral immune response, are located in lymph nodes and lymphatic structures adjacent to the TME [33]. Their primary functions include antibody production, antigen presentation, and cytokines secretion. Their involvement in the formation of tertiary lymphoid structures is a favorable prognostic indicator in several cancers, as it promotes cytotoxic immune responses [34]. However, their presence can also be associated with unfavorable outcomes in other tumor types, particularly by promoting cellular phenotypes that inhibit anti-tumor immunity [9,35].

T-lymphocytes (TLs), derived from the same precursor as B cells, feature a T-cell receptor (TCR) that recognizes antigens presented by the major histocompatibility complex. TLs are divided into CD8+ cytotoxic and CD4+ helper cells, with several subtypes having either anti-tumor or pro-tumor effects [36]. CD8+ TLs target cancer cells and inhibit angiogenesis, while CD4+ TH2 cells support B lymphocytes [37,38,39]. Conversely, NK cells are particularly effective against circulating tumor cells but can also promote tumorigenesis in the TME [40]. CD4+ TH17 cells promote antimicrobial inflammation, whereas regulatory T cells (Tregs) suppress anti-tumor immune responses, with their reduction potentially leading to metastatic regression in certain cancers [29,41,42].

Neutrophils, comprising the majority of circulating leukocytes, are essential for the adaptive immune defense [43]. Their behavior within the TME can either suppress or promote tumor growth, depending on the type and stage of cancer. Initially, neutrophils stimulate inflammation by releasing cytokines and reactive oxygen species, inducing tumor cell apoptosis. However, they can also have pro-tumor effects, particularly through the release of various enzymes and the induction of immunosuppression [16].

#### 2.3.3. Extracellular Matrix

The ECM is a complex network of macromolecules, including glycoproteins, collagen, and enzymes, that supports biomechanical activities. This acellular structure consists of active tissue components that influence cellular processes such as adhesion, proliferation, and communication [44]. The ECM can limit cancer development in its early stages but can also promote tumor progression. Its composition serves as a predictive factor for cancer progression; tumors with high expression of protease inhibitors are associated with a good prognosis, while those with significant expression of integrins and matrix metalloproteinases (MMPs) are characterized by a poor prognosis [45].

#### 2.3.4. Adipocytes

Adipocytes are specialized cells that play a role in regulating the energy balance by storing energy in the form of fat (Figure 3). They influence the TME by secreting a wide range of metabolites, enzymes, hormones, growth factors, and cytokines. Adipocytes maintain a dynamic and reciprocal relationship with tumor cells, thereby promoting their progression [46]. Additionally, adipocytes modify the ECM by secreting metalloproteinases [47].

#### 2.3.5. Fibroblasts

Fibroblasts are a highly abundant and multifunctional cell type found in connective tissue. They produce ECM and cellular membrane components [48]. Cancer-associated fibroblasts (CAFs) present in the TME are distinct from normal fibroblasts and are significantly more numerous (Figure 3). This particular cell type will be discussed in detail in Section 3.1 below.

## 3. Cancer-Associated Fibroblasts and Fibroblast Activation Protein

### 3.1. Role and Properties of Cancer-Associated Fibroblasts

#### 3.1.1. Morphology and Subtypes

CAFs form a heterogeneous group of activated fibroblasts with multiple functions, making them difficult to define. They do not express conventional epithelial, endothelial, or leukocyte markers and are characterized by an elongated morphology [49]. CAFs cannot be distinguished from normal fibroblasts under an electron microscope and are often identified by their expression of αSMA [48]. Other markers, such as fibroblast activation protein (FAP) and the platelet-derived growth factor (PDGF) receptor, have been correlated with a poor prognosis, but none are exclusive to CAFs. Additionally, CAFs exhibit significant heterogeneity, both within a single type of tumor tissue and across different cancer types. They are often classified into three subtypes: myCAFs (myofibroblasts), iCAF (inflammatory), and apCAF (antigen-presenting). MyCAFs are characterized by αSMA expression, ECM proteins secretion, and activation by TGF-β. Conversely, iCAFs secrete pro-inflammatory cytokines and are differentiated by IL-1β. The less common apCAFs have the ability to present antigens on their surface [50]. Four CAF subpopulations (S1–S4) can also be differentiated by their location: S1 is found in tumors and inflammatory diseases, S4 only in tumors, and S2 and S3 in peri-tumoral regions [51].

**Figure 4:**
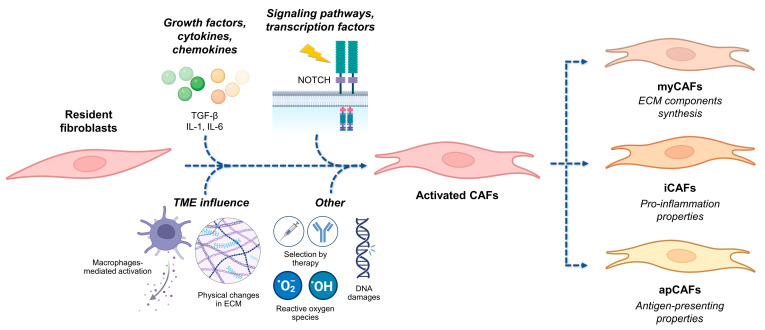
Main transformation mechanisms from resident fibroblasts to cancer-associated fibroblasts.

CAFs primarily originate from resident fibroblasts adjacent to the tumor or from mesenchymal cells recruited to the tumor mass and activated by cancer cells (Figure 4). Several factors play a role in fibroblast activation, including TGF family ligands, the Notch signaling pathway, and inflammatory signals such as interleukins 1 and 6 [52,53]. Similarly, the TME contributes to CAF activation, particularly through macrophages and physical changes in the extracellular matrix, such as fibroblast stretching [54,55,56]. Therapies can also induce CAF transformations: cytotoxic chemotherapies, targeted therapies, and radiotherapy can promote CAF activation. This transformation is often associated with mechanisms of treatment resistance. Therefore, targeting CAFs in therapy could provide a strategy to overcome resistance to conventional treatments [49,57].

#### 3.1.2. Cancer Predisposition Properties

Phenotypically modified fibroblasts may play a role in cancer predisposition. According to the work of Kopelovich, skin fibroblasts from patients highly predisposed to breast cancer exhibit an abnormal phenotype, including a reduced serum requirement for proliferation [58]. Similarly, skin fibroblasts taken from patients with breast cancer, malignant melanoma, retinoblastoma, and Wilms’ tumors show an increased proliferation rate in vitro, suggesting that alterations in fibroblast behavior could promote cancer initiation [59].

#### 3.1.3. Immune Modulating Properties

Similar to normal fibroblasts, which trigger the secretion of immune factors following tissue injury, CAFs generate cytokines and chemokines, predominantly with immunosuppressive properties [60]. Notably, TGF-β induces the expression of PD-1 in tumors, leading to immune system suppression, as previously mentioned. More specifically, it has been shown that FAP-dependent activation pathways contribute to the blockage of immune checkpoints in colorectal cancer cells [61]. In addition, antigens presented by apCAFs result in the activation of CD4+ T cells and the suppression of CD8+ T cells [62]. CAFs also influence MDSCs through FAP, TGF-β, or interleukin-6 [63,64].

#### 3.1.4. Extracellular Matrix Remodeling Properties

The ECM, as previously discussed, serves as a mechanical scaffold for tissue structuring. CAFs secrete type I collagen, a fundamental component of the ECM [65,66]. Additionally, type IV collagen is upregulated by CAFs, leading to the recruitment of macrophages, increased inflammation, and enhanced angiogenesis [67]. Fibronectin, involved in tumor invasion, is also secreted by CAFs. MMPs produced by fibroblasts contribute to the stiffening of tumor tissue and the formation of pathways that facilitate cancer cell invasion [68]. This stiffness is a marker of cancer progression, influencing tumor cell proliferation and resistance to treatments [69]. ECM remodeling and the creation of invasion pathways by CAFs also rely on the production of cell receptors, such as integrins, and their connection to the actin cytoskeleton [70].

#### 3.1.5. Tumor Growth and Invasion-Promoting Properties

Through similar mechanisms to those affecting immunity, CAFs also stimulate tumor growth via growth factors and cytokines secretion [71]. For instance, VEGF induces microvascular permeability, leading to the extravasation of plasma proteins such as fibrin, which triggers an influx of fibroblasts, inflammatory cells, and endothelial cells [72]. These cells contribute to the production of an ECM rich in fibronectin and type I collagen, promoting tumor angiogenesis. CAFs also produce stromal cell-derived factor 1 (SDF-1 or CXCL12), which is involved in both the recruitment of endothelial progenitors to the tumor and in cancer cell growth [73].

During metastasis, cancer cells separate from the primary tumor mass. The invasion pathways created by CAFs facilitate the dissemination of tumor cells, enabling their disengagement from the primary tumor [74]. Regarding growth factors, TGF-β promotes inflammation and enhances the metastatic potential of cancer cells by inducing EMT in epithelial cells, granting them mesenchymal properties [75]. Other metastasis-related factors secreted by CAFs and involved in EMT have been extensively studied, including IL-6, osteopontin, hepatocyte growth factor (HGF), and CXCL12 [76,77]. Activation of the PDGF receptor on CAFs promotes cancer cell survival and is associated with increased invasion and poor prognosis in several cancers [78,79]. CAFs also indirectly stimulate metastasis through their interactions with TAMs: several CAFs-released cytokines recruit TAMs, such as CCL2 and CCL5 (chemokine ligand 2/5), while IL-4 and IL-6 polarize TAMs toward the M2 phenotype, which supports tumor growth and metastasis [80,81,82,83]. Lastly, CAFs express tenascin and periostin within the ECM, which are correlated with invasion, angiogenesis, and metastatic potential [84,85].

#### 3.1.6. Treatment Resistance

CAFs can induce resistance to therapies through various mechanisms related to their intrinsic properties. For instance, ECM remodeling can create a physical barrier that hinders the penetration of chemotherapeutic agents into the tumor. Hypoxia further reduces the sensitivity to anticancer drugs [86,87]. As previously mentioned, HGF secreted by CAFs can confer resistance to targeted therapies such as dabrafenib in BRAF-mutated melanoma cells [88]. Similarly, the efficacy of antiangiogenic treatments is often limited, potentially due to the production of pro-angiogenic factors by CAFs [89]. Additionally, CAF-induced immunosuppression may decrease the effectiveness of immune checkpoint inhibitors, such as anti-CTLA-4, anti-PD-1, and anti-PDL-1 therapies. In this context, inhibiting CXCL12 produced by CAFs has been shown to resensitize pancreatic cancer cells to anti-PDL-1 therapy [90]. Another study demonstrated that CAF-mediated activation of the JAK-STAT signaling pathway could confer chemotherapy resistance to gastric cancer cells, while IL-6 secreted by CAFs could protect gastric cancer cells through a paracrine signaling pathway [91].

**Figure 5:**
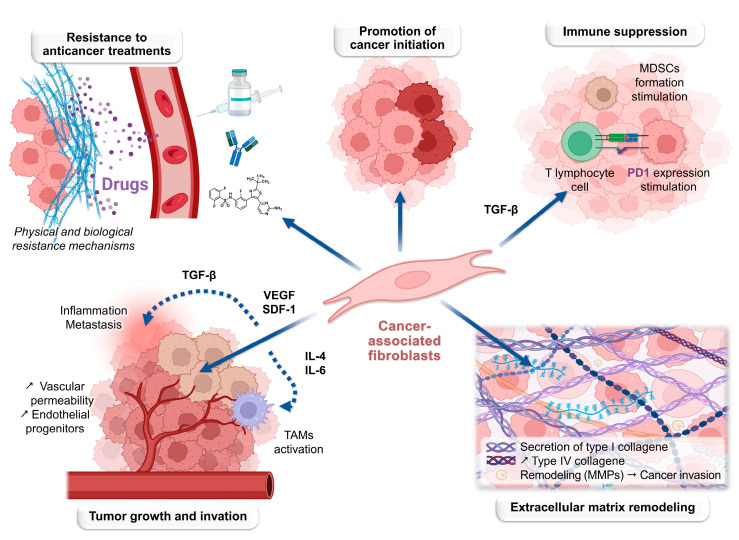
Summary of CAFs’ general properties.

Exosomes are small vesicles secreted by various cell types and contain multiple signaling molecules. CAFs produce exosomes that transmit genetic information, thereby promoting chemoresistance, as observed in pancreatic cancer, where gemcitabine-treated CAFs generate exosomes that increase chemoresistance [92]. The differentiation of cancer cells into stem-like cells, which are capable of dedifferentiation, self-renewal, and chemotherapy drug efflux, is another resistance mechanism associated with CAFs [93]. Finally, CAFs influence chemotherapy resistance in certain leukemias by acting on the cell cycle, with growth differentiation factor 15 (GDF15) blocking the cell cycle in the G0/G1 phase, thus limiting chemotherapy sensitivity [94]. Figure 5 summarizes the various effects exerted by CAFs, particularly in cancer pathologies.

### 3.2. Structure, Expression, and Activities of the Fibroblast Activation Protein

FAP was first described in 1986 by Rettig et al., who named it based on its expression by fibroblasts, particularly CAFs [95]. Simultaneously, a serine protease was identified by Aoyama and Chen in melanoma cells; it was termed seprase due to its enzymatic activity [96]. It was not until 1997 that gene sequencing revealed that FAP and seprase referred to the same protein [97]. Overall, FAP is a type II transmembrane serine protease with a molecular weight of 97 kDa, belonging to the dipeptidyl peptidase (DPP) family and related to the endopeptidase prolyl oligopeptidase (PE). It shares approximately 50% homology with dipeptidyl peptidase 4 (DPP IV) [97,98].

#### 3.2.1. Fibroblast Activation Protein Structure

**Figure 6:**
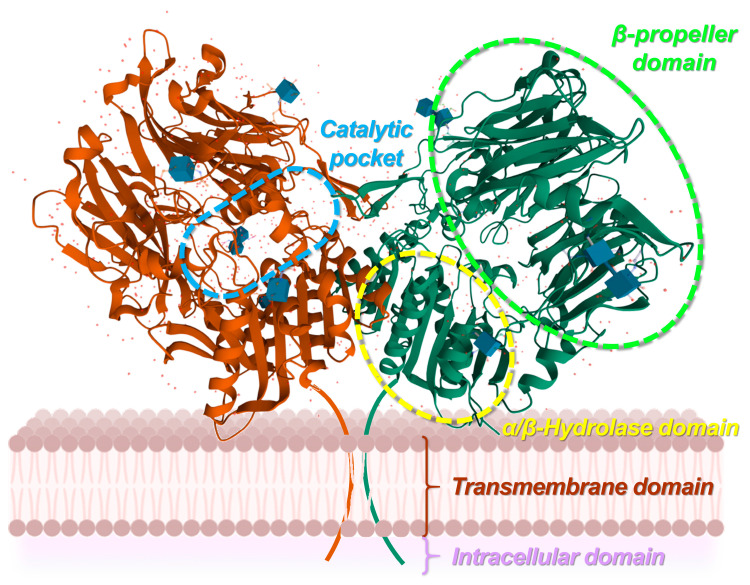
Schematic representation of transmembrane FAP based on the crystal structure of human FAP alpha; PDB 1Z68 [103].

FAP is a protein with a primary structure consisting of 760 amino acids, where residues 1–4 form the intracellular domain, 5–25 make up the transmembrane domain, and 26–760 compose the extracellular domain (Figure 6) [99]. The extracellular region includes a beta-propeller domain formed by eight blades (each comprising three or four beta sheets), which confers substrate selectivity to the enzyme, as well as an alpha/beta hydrolase domain [100]. Serine proteases, including FAP and DPP IV, contain a catalytic triad made up of a serine, aspartic acid, and histidine. The serine acts as a nucleophile, cleaving N-terminal proline (Pro)-X peptide bonds, where X can be any amino acid except proline or hydroxyproline, a characteristic of dipeptidyl peptidase activity. FAP exhibits dipeptidyl peptidase activity similar to DPP IV, and like PE, it also displays endopeptidase activity, which preferentially targets glycine (Gly)-Pro-X motifs [101]. This endopeptidase activity is central to both specific detection methods for FAP and the design of its specific inhibitors [100]. FAP monomers do not exhibit enzymatic activity; however, they assemble into active homodimers or heterodimers with DPP IV [102].

Of note, FAP exists in a soluble form known as antiplasmin-cleaving enzyme (APCE), which circulates in human plasma without the intracellular and transmembrane regions [99]. Structurally, soluble FAP contains the same two domains as its transmembrane counterpart: an eight-bladed beta-propeller domain and an alpha/beta hydrolase domain. This structure also features a large cavity with a catalytic triad composed of the residues Ser624, Asp702, and His734. The active site cavity is only accessible to elongated peptides or unfolded protein fragments, giving soluble FAP a degree of specificity [104]. This form of FAP also exhibits both dipeptidyl peptidase and endopeptidase activities. It is produced in various contexts, including by reactive stromal fibroblasts during wound healing, and in some healthy tissues such as placenta, uterine stroma during the proliferative phase, embryonic tissues, and multipotent stromal cells from bone marrow [105].

#### 3.2.2. Expression and Overexpression of FAP

In the context of cancer diseases, the FAP overexpression by CAFs is well established, but this is also true for mesenchymal stem cells, sarcoma and melanoma cells, M2 TAMs, and adipocytes within the MET [102]. Under pathological conditions, FAP expression is significantly elevated in the tumor stroma of breast, lung, colorectal, prostate, gastric, pancreatic, thyroid, cervical, and urothelial cancers [106]. This increased expression makes FAP a relevant target for molecular imaging techniques in oncology. Overall, research data suggest that FAP can be considered an independent poor prognostic factor for multiple cancer types and is associated with reduced overall survival in various malignancies [107,108,109]. However, in certain cancers, such as breast cancer, CAF-S4 cells show little or no FAP expression but are still associated with the development of metastases, suggesting that FAP is not the sole pro-tumor mechanism related to CAFs [110,111].

FAP is also expressed in liver fibrosis and cirrhosis, which could be attributed to its role in wound healing processes. Elevated levels of FAP are correlated with the stage of fibrosis, suggesting its potential value as a biomarker for fibrotic diseases [112]. Furthermore, it is overexpressed in Crohn’s disease, an autoimmune disorder characterized by chronic inflammation and intestinal fibrosis. Its presence in chronic inflammatory bowel diseases is therefore consistent [113]. Lastly, FAP and APCE are found in rheumatoid arthritis and osteoarthritis, where they are associated with joint inflammation, cartilage degradation, and disease severity [114,115].

#### 3.2.3. Enzymatic Activities of FAP

Active FAP adopts a dimeric configuration within the cell membrane, requiring a specific region in its transmembrane domain for this association [116]. This sequence consists of three small amino acid residues (glycine, serine, and alanine), each spaced by three variable residues (composed of valine, alanine, threonine, and/or leucine). Mutations affecting these residues disrupt FAP monomer association, leading to decreased enzymatic activity and intracellular accumulation of mutant FAP. These findings suggest a connection between FAP dimerization and its cellular localization [117]. Additionally, studies support the hypothesis that, once integrated into the plasma membrane, FAP concentrates within invadopodia (tumor cell protrusions) [118,119].

**Figure 7:**
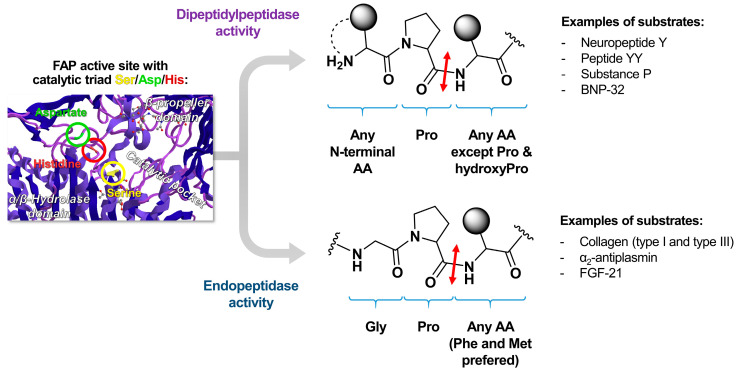
Schematic representations of enzymatic activities catalyzed by FAP, with the red arrow indicating the peptide bond cleaved by enzymatic action. Stereochemistry of the amino acids is not shown in this representation. AA: amino acid; Ser: serine; Asp: aspartic acid; His: histidine; Pro: proline; hydroxyPro: hydroxyproline; Gly: glycine; Phe: phenylalanine; Met: methionine; BNP-32: brain natriuretic peptide 32; FGF-21: fibroblast growth factor 21.

The dual enzymatic activity of FAP offers a variety of potential substrates. Its endopeptidase activity allows to cleave denatured type I and type III collagen, which frequently contain Gly-Pro dipeptide sequences [120]. Through this activity, FAP-expressing CAFs can remodel the ECM by cleaving collagen and altering bioactive signaling peptides in cancer. Endopeptidase activity also facilitates the cleavage of α-2 antiplasmin (inhibitor of fibrinolysis) and fibroblast growth factor 21 (FGF 21) [121,122]. Simultaneously, the dipeptidyl peptidase activity enables the cleavage of neuropeptide Y, peptide YY, substance P, and brain natriuretic peptide 32, which are involved in regulating food intake, satiety, pain, and the renin–angiotensin–aldosterone system, respectively [123]. Figure 7 illustrates FAP’s substrates according to its enzymatic activity and the corresponding cleaved bond.

CAFs overexpression of FAP leads to significant modulation of the secretome, characterized by increased secretion of proliferative, inflammatory, and ECM remodeling factors [124]. Tumor mesenchymal cells expressing FAP show enhanced adhesion and migration on substrates such as fibronectin or type I and type IV collagen [125]. In gastric cancers, FAP expression by tumor epithelial cells is associated with increased cell proliferation, highlighting the role of FAP in tumor progression [126]. Additionally, FAP appears to modulate immune responses, as it has been linked to the suppression of CD4+ T-lymphocyte proliferation and the promotion of cellular senescence [127]. Regarding chemotherapy resistance, the introduction of FAP into ovarian tumor cells has been associated with reduced sensitivity to cisplatin, a first-line cytotoxic agent for this cancer [128]. Furthermore, coculture with FAP+ mesenchymal stem cells has been observed to enhance the survival of myeloma cells treated with bortezomib [129].

It has been proposed that FAP may exhibit non-enzymatic activity, which could also trigger the effects previously mentioned [127]. To demonstrate this, catalytically inactive FAPs were studied by Baobei Lv’s team. In this work, breast cancer cells transfected to express FAP showed increased growth, adhesion, and migration, even when enzymatic activity was suppressed. Both wild-type and mutant FAPs caused comparable increases in cancer-related signaling pathways and the expression of matrix metalloproteinase 9 (MMP9), suggesting that FAP might exert intrinsic effects on cellular signaling independently of its enzymatic activity [130].

## 4. Fibroblast Activation Protein Inhibitors as Imaging Agents in Nuclear Medicine

### 4.1. Design and Development of Quinoline-Based FAP Inhibitors

**Figure 8:**
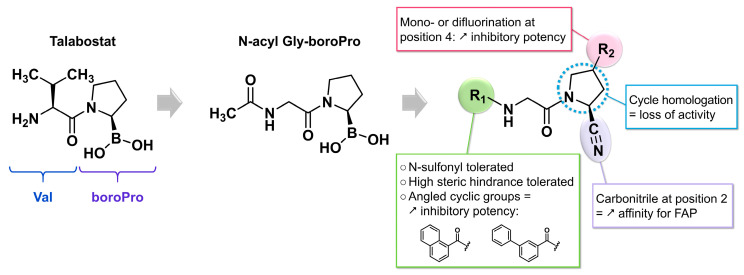
Chemical structure of talabostat with design rationale for the initial FAPI derivatives and first SARs.

As an enzyme overexpressed at the extracellular membrane of CAFs, FAP is highly specific to the TME and represents an ideal target for molecular imaging. In this context, FAP inhibitors (FAPIs) have been developed to design imaging agents that can be easily labeled with beta-plus emitting radiometals for PET imaging. Early works were inspired by talabostat, a prolyl-peptidase inhibitor with a valinyl-L-boroproline (Val-boroPro) motif that mimics the NH_2_-X-Pro motif recognized by FAP’s catalytic site (Figure 8). By comparing the active site of FAP to that of DPP IV, a general N-acyl Gly-boroPro structure was proposed for inhibitors, based on the substrate preferences of FAP’s endopeptidase activity. This study also identified inhibitors based on the N-acyl-Gly-Pro motif as promising FAPI candidates [120].

A team from the University of Antwerp built on this work to propose a similar inhibitor structure, simply replacing the carboxylic acid group of proline with a nitrile function. In this study, two types of modifications were examined on the initial molecular framework: first, modulation of the N-acyl substituent (R1), followed by modifications to the 2-cyanopyrrolidine component (R2). The in vitro efficacy and selectivity results obtained with these derivatives suggest the potential of an aromatic azaheterocyclic group attached to the N-acyl. Notably, only compounds with such a group showed significant inhibition of the FAP enzyme, without this effect being related to their ability to bind to prolyl endopeptidase (PE), a close relative of FAP. From the 27 compounds synthesized and evaluated in this series, structure–activity relationships (SARs) were established (Figure 8). Replacing the N-acyl group with a N-sulfonyl group did not alter the selectivity for FAP compared to PE. The steric hindrance of N-acyl substituents did not interfere with the binding of the studied ligands to FAP. Compounds with azaheterocyclic parts misaligned relative to the acyl group, such as bicyclic or biaryl motifs, demonstrated improved inhibitory potency. At the pyrrolidine core, a carbonitrile adjacent to the nitrogen atom increased the affinity for FAP. This observation can be explained by an interaction between the hydroxyl group of a serine in the enzyme’s active site and the carbon of the carbonitrile, potentially involving the formation of a covalent carboximidate bond with the enzyme [131]. Another study observed that such FAP inhibitors bind very tightly to the active site of FAP, resulting in the practically irreversible character of the compounds. This supports the idea of a covalent interaction between the carbonitrile and the serine residue of the FAP catalytic site [132]. Substitution at position 4 of the 2-cyanopyrrolidine ring was then explored. Fluorinated inhibitors were the only compounds that outperformed their unsubstituted analogs, with no significant difference in median inhibitory concentration (IC_50_) observed between mono- and di-fluorinated compounds. Finally, replacing the 2-cyanopyrrolidine ring with a homologous 2-cyanopiperidine ring (enlarged by one methylene unit) led to a loss of affinity for both FAP and PE, suggesting that the catalytic pocket of these enzymes has limited space (Figure 8) [131].

**Figure 9:**
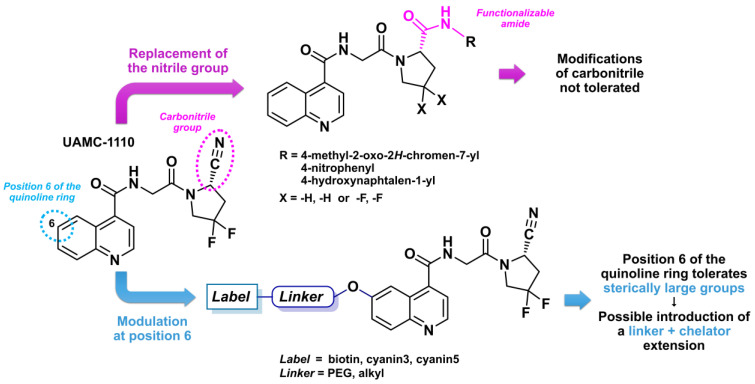
Chemical structure of UAMC-1110 and design rationale for chelator-containing FAPI imaging agents [132,134].

The FAP inhibitor UAMC-1110 (Figure 9), first described in 2014, was one of the earliest small molecules to exhibit a high affinity for FAP (in the nanomolar range) combined with significant selectivity over prolyl-endopeptidase (PREP) and DPP enzymes. Additionally, this molecule demonstrated a favorable pharmacokinetic profile, including high oral bioavailability and excellent plasma stability. It incorporates the previously established SAR elements, featuring a carbonitrile and two fluorine atoms on the pyrrolidine ring, and is based on a quinoline azaheterocycle [133]. In 2019, a more detailed SAR study was conducted using derivatives of UAMC-1110. Specifically, the replacement of the carbonitrile group with an amide, to enable the addition of substituents on the pyrrolidine ring, was explored. However, such modifications were not well tolerated. Furthermore, it was confirmed that difluorination of the pyrrolidine residue enhanced the affinity for FAP, though this came at the cost of increased lipophilicity and reduced water solubility [134]. In 2021, Van Rymenant et al. continued this work, demonstrating that the addition of bulky chemical groups at position 6 of the quinoline core did not alter the FAP affinity [132]. This tolerance suggested the possibility of incorporating a linker and chelator in this scaffold.

**Figure 10:**
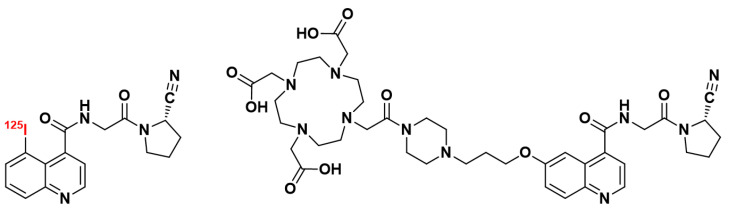
Chemical structures of FAPI-01 (**left**) and FAPI-02 (**right**).

In 2013, a research team from the University of Antwerp focused on 2-cyanopyrrolidine derivatives, which had previously been explored in the design of DPP IV inhibitors, also known as gliptins. They concentrated on a group of 2-cyanopyrrolidines that represent the first examples of inhibitors combining a strong affinity (in the nanomolar range) for FAP with significant selectivity against PE and DPP. Talabostat and linagliptin were used as reference compounds. This study confirmed the structure N-(4-quinolinyl)glycyl-(2-cyanopyrrolidine), featuring a Gly-cyanoPro motif, as highly promising for the discovery of FAP inhibitors [135], also highlighting the essential role of the quinoline ring in obtaining high-affinity FAPI compounds [133]. Loktev’s team built upon the N-(4-quinolinyl)glycyl-(2-cyanopyrrolidine) structure to design the first FAPI compounds used in nuclear medicine: FAPI-01 and FAPI-02 (Figure 10) [136].

FAPI-01 is a quinoline derivative substituted at position 5 by an iodine-125 atom. This gamma-emitting isotope with a long half-life (59.4 days) is particularly useful in biodistribution studies. FAPI-01 selectively bonds human and murine FAP and is rapidly internalized in FAP-expressing cells but exhibits time-dependent efflux. It is also prone to deiodination. To prevent the rapid activity loss of [^125^I]I-FAPI-01 due to enzymatic deiodination, a non-halogenated derivative was developed: FAPI-02. In this compound, the FAP-binding moiety is functionalized with a spacer bearing a DOTA chelator [136]. The use of ^68^Ga-radiolabeled FAPI-02 in PET imaging was initially studied in three patients with metastatic cancers (lung, breast, and pancreas). In all three patients, [^68^Ga]Ga-FAPI-02 showed significant accumulation in tumors and metastases (maximal standardized uptake value [SUVmax] = 13.3), with no accumulation in non-cancerous tissues (SUVmax = 3.6), and was rapidly cleared by the kidney (SUVmax = 6.1). Its ^177^Lu-radiolabeled counterpart FAPI-02 was rapidly internalized into FAP-expressing cells and exhibited high tumor uptake in mice bearing HT-1080-FAP (epithelial) or SK-LMS-1 (vulvar) cell xenografts. No significant accumulation in normal tissues was observed, and blood clearance was rapid, allowing highly contrasted SPECT imaging.

**Figure 11:**
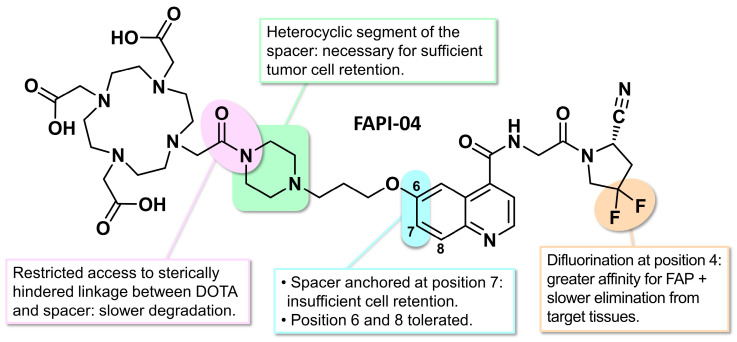
Chemical structure of FAPI-04 and initial SARs in the quinoline series.

The research team who developed the first quinoline-structured FAPI compounds previously discussed also designed analogs ranging from FAPI-03 to FAPI-15 and explored their SARs in detail. In this series, FAPI-04 emerged with the best potential for clinical use, both for diagnostic and therapeutic applications (Figure 11). Like its analog FAPI-02, FAPI-04 exhibited rapid internalization into FAP-positive tumors and fast renal clearance of its unbound fraction, leading to swift accumulation at tumor sites. As previously noted, difluorination of the pyrrolidine ring enhanced the FAP affinity. This radiotracer showed a greater affinity for FAP than for DPP IV and displayed a slower elimination rate from target tissues [137]. Similar to [^68^Ga]Ga-FAPI-02, [^68^Ga]Ga-FAPI-04 exhibited strong accumulation in primary tumors and metastases (SUVmax = 23.86), with minimal retention in healthy tissues (SUVmax = 2.35) at 1 h post-injection (p.i.) in a patient with breast cancer.

Although FAPI-04 offered the best potential, other synthesized FAPI variants helped establish SARs. For example, derivatives with a simple 3-amino-1-propyl spacer (FAPI-06 and FAPI-07) showed adequate cellular binding at 1 and 4 h but were almost completely cleared after 24 h. This suggests that the heterocyclic segment of the spacer is necessary for sufficient tumor cell retention. The linkage between DOTA and propylamine (FAPI-06 and 07) is much more accessible than that between DOTA and piperazine (FAPI-02 and 04), which may lead to more rapid enzymatic degradation. Modifications where the spacer is attached at position 7 (instead of position 6) of the quinoline ring (FAPI-08 and FAPI-09) exhibited faster elimination from target cells during incubation. This indicates that the position of the spacer on the quinoline ring is another critical factor, with position 6 being preferred, although position 8 was also explored in the subsequent series [138]. FAPI-10, featuring a nuclear localization signal (a short targeting sequence enabling transport from the cytoplasm to the nucleus) in its spacer structure [139], accumulated in the kidneys and was therefore not clinically useful. Overall, comparison of the 11 synthesized FAPI compounds revealed that, in addition to difluorination of the pyrrolidine ring, other structural prerequisites are necessary for optimal tumor retention, such as the position of the spacer on the quinoline ring and the nature of the spacer itself (Figure 11) [140].

**Figure 12:**
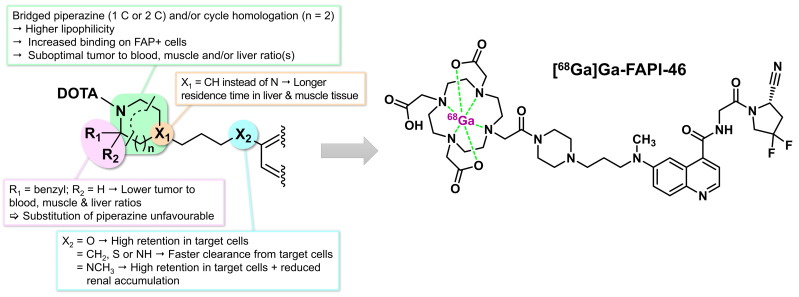
Extended SARs and chemical structure of [^68^Ga]Ga-FAPI-46.

The same team conducted additional studies to optimize the tumor retention of FAPI-04. To this end, compound lipophilicity was modulated by varying the spacer region, primarily through bridged or substituted analogs of the original piperazine portion or by altering the chemistry used to attach the DOTA or the spacer to the quinoline moiety (Figure 12) [141]. In an effort to improve target binding, the electronic density of the atom anchoring the spacer to the quinoline ring was adjusted, which influenced the proton acceptance capacity. Specifically, the ether-oxide initially used as the anchor point for the spacer was replaced with methylene, sulfide, amine, and methylamine groups. These modifications, along with those made to the piperazine fraction or the spacer region, did not significantly influence the half-maximal inhibitory concentrations (IC_50_). However, they had substantial effects on in vitro efflux kinetics. More specifically, FAPI-21 (with an ether-oxide link and a bridged piperazine) and FAPI-46 (with a tertiary amine to anchor its spacer) were eliminated more slowly from cells than FAPI-04, even though FAPI-46 shared the same DOTA-piperazine structure as FAPI-04. On the other hand, FAPI-39, 40, and 41 (containing other anchoring functions) were rapidly cleared from FAP-positive cells, highlighting the influence of the type of linkage between the spacer and the quinoline ring on this parameter. In initial in vivo PET imaging studies conducted on HT-1080-FAP xenografted mice, FAPI-21, 36, 46, and 55 demonstrated better tumor uptake but also higher muscular activity, resulting in lower-quality images. Among these, FAPI-55 (with a piperidine instead of piperazine) had the best tumor absorption, but its lipophilicity also led to prolonged hepatic residence time. Ultimately, FAPI-46 was identified as the best theranostic agent in this series (Figure 12), characterized by the most favorable tumor-to-healthy tissue ratio.

Among the previously discussed derivatives, FAPI-21 (with ether anchor and bridged piperazine) and FAPI-46 were identified as having the highest clinical potential, a finding confirmed through early PET studies in eight patients with metastatic mucoepidermoid, oropharyngeal, ovarian, or colorectal carcinoma. Compared to FAPI-04, both FAPI-46 and FAPI-21 radiolabeled with ^68^Ga demonstrated slightly better tumor-to-healthy tissue ratios (SUVmax tumor for FAPI-21 = 11.93; FAPI-46 = 12.76; FAPI-04 = 10.07). However, FAPI-21 also exhibited particularly intense uptake in the oral mucosa (SUVmax = 3.38), salivary glands (SUVmax = 3.69), and thyroid (SUVmax = 3.25), the phenomenon remaining unexplained by the study authors. In comparison, FAPI-46 showed lower SUVmax values for the oral mucosa (SUVmax = 1.49), salivary glands (SUVmax = 1.38), and thyroid (SUVmax = 2.25), suggesting potentially lower toxicity in therapeutic applications compared to FAPI-21 [141]. Table 1 summarizes the main properties of the quinoline FAPIs discussed in this review.

### 4.2. Clinical Use of FAPI-04 and FAPI-46 in Oncology

A significant number of clinical studies suggest that imaging with ^68^Ga-labeled FAPI-04 or FAPI-46 could serve as an alternative to [^18^F]FDG PET in certain oncological indications, particularly for thyroid, liver, and biliary tract cancers, as well as peritoneal carcinomatosis. These cancers are characterized by a low uptake of [^18^F]FDG. For several other cancers, such as breast, ovarian, gastric, pancreatic, and bladder cancers, [^18^F]FDG uptake is inconsistent. However, for cancers like lung, head and neck, or colorectal cancer, where [^18^F]FDG tumor uptake is high, the diagnostic benefit of FAPI could be more limited [142]. This section will therefore focus on cancers with low [^18^F]FDG uptake.

#### 4.2.1. Liver and Biliary Tract Cancers

**Figure 13:**
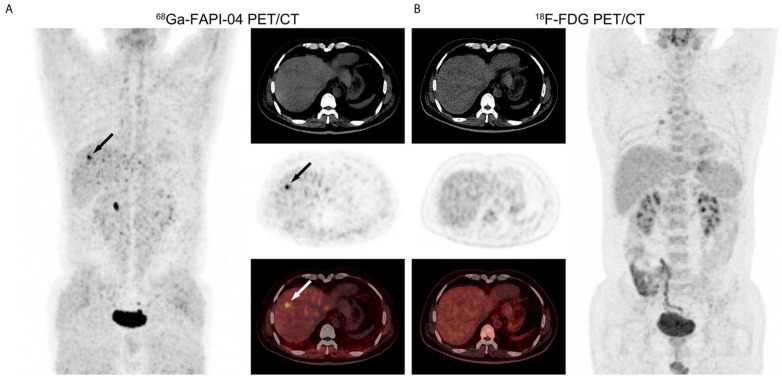
PET/CT scans of a 53-year-old male diagnosed with moderately differentiated HCC. (**A**) The [^68^Ga]Ga-FAPI-04 PET/CT detected a high-affinity lesion (black and white arrows, SUVmax = 7.36) in the liver’s right lobe. (**B**) In contrast, no abnormalities were identified in the liver on the [^18^F] FDG PET/CT images (SUVmax = 2.36). Images originally published by Wang et al. [152].

Liver cancer is the fourth leading cause of death worldwide, with over 800,000 deaths per year [143]. The most commonly diagnosed type is hepatocellular carcinoma (HCC), which originates from hepatocytes, followed by cholangiocarcinoma (CC), arising from bile duct cells [144,145]. [^18^F]FDG is taken up by HCC cells in only 60% of cases, and most well-differentiated HCCs are negative on [^18^F]FDG PET scans (Figure 13) [146]. Similarly, the role of [^18^F]FDG in diagnosing CC is debated. In the study by Petrowsky et al., PET showed no significant advantage over contrast-enhanced computed tomography in diagnosing extrahepatic CC and intrahepatic lesions [147]. In a prospective study of 41 patients suspected of having HCC or CC, Rajaraman et al. found that [^68^Ga]Ga-FAPI-04 outperformed [^18^F]FDG PET with a sensitivity, specificity, and accuracy of 96.8% vs. 51.6%, 90% vs. 100%, and 95.1% vs. 63.4%, respectively [148]. These findings are supported by numerous other studies, particularly for [^68^Ga]Ga-FAPI-46 [149,150,151]. For instance, a retrospective study by Siripongsatian et al. compared tumor detection rates using MRI, [^18^F]FDG PET, and [^68^Ga]Ga-FAPI-46 PET in 27 patients with CC or HCC; the same intrahepatic lesions detected by MRI were also identified by [^68^Ga]Ga-FAPI-46 PET (100% sensitivity), whereas [^18^F]FDG PET had a sensitivity of only 58% [149].

#### 4.2.2. Recurrent Well-Differentiated Thyroid Cancer

**Figure 14:**
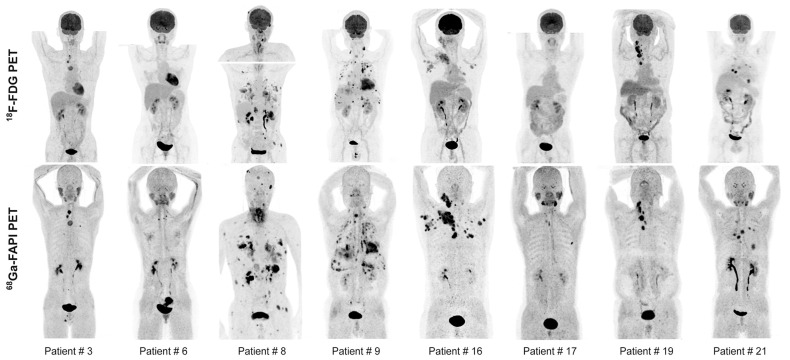
[^18^F]FDG and [^68^Ga]Ga-FAPI-04 PET/CT images of 8 patients with metastatic differentiated thyroid cancer. ^68^Ga-FAPI PET/CT demonstrated superior detection compared to ^18^F-FDG PET/CT for identifying local recurrence (patient 9); cervical lymph node metastases (patients 6, 16, and 17); mediastinal lymph node metastases (patients 8, 16, 17, and 21); axillary lymph node metastases (patient 16); abdominal lymph node metastases (patient 9); lung metastases (patients 3, 9, 16, and 21); subcutaneous metastases (patient 8); and pleural metastases (patient 9). Images originally published by Fu et al. [155].

[^18^F]FDG PET imaging plays a role in evaluating recurrent differentiated thyroid cancer (DTC) in patients with thyroglobulin-elevated negative iodine scintigraphy [153]. However, these patients are challenging to diagnose and treat, as [^18^F]FDG PET shows variable sensitivity (ranging from 68.8% to 82%) and can result in false negatives in 8% to 21.1% of cases [154]. In this context, studies have explored the potential of FAPI PET imaging in such cancers. Fu et al. demonstrated the superiority of [^68^Ga]Ga-FAPI-04 PET over FDG PET in 35 patients with metastatic DTC, showing higher SUVmax values (7 vs. 4) and improved sensitivity (83% vs. 65% for neck lesions and 79% vs. 59% for distant metastases) [155]. The key images from this study are presented in Figure 14. Another retrospective study involving 29 patients with recurrent papillary thyroid carcinoma also demonstrated the superiority of [^68^Ga]Ga-FAPI-04 PET over [^18^F]FDG PET in detecting recurrence in DTC patients (86% vs. 72.4%) [156].

#### 4.2.3. Sarcomas

Soft tissue sarcomas are rare malignant tumors that develop in the extra-skeletal connective or supporting tissues of the body, such as adipose, muscular, vascular, fibrous tissues, and the peripheral nervous system [157]. According to a meta-analysis of 15 prospective and retrospective studies, [^18^F]FDG PET is useful in diagnosing high- or intermediate-grade sarcomas, with a typical SUV of 2 or higher. However, low-grade tumors are more difficult to diagnose and differentiate from benign tumors, as they often show SUVs below 2 [158]. A prospective study involving 45 patients previously diagnosed with recurrent soft tissue sarcoma explored the potential benefits of FAPI PET imaging compared to [^18^F]FDG for detecting lesions. [^18^F]FDG PET detected about two-thirds of recurrent lesions, with a sensitivity of 65.96% and a specificity of 21.43%, whereas [^68^Ga]Ga-FAPI-04 PET identified almost all lesions (275 out of 282) and showed significantly higher sensitivity and specificity (97.52% and 60.71%, respectively) [159]. Another prospective study focused on [^68^Ga]Ga-FAPI-46 established a correlation between histopathological FAP expression and the intensity of [^68^Ga]Ga-FAPI-46 uptake in bone and soft tissue sarcomas in 47 patients with sarcomas. It also compared [^68^Ga]Ga-FAPI-04 PET with [^18^F]FDG PET in 43 of these patients. The exams revealed differences in disease staging, with six patients being reclassified from locoregional to metastatic disease after undergoing [^68^Ga]Ga-FAPI-04 PET [160].

#### 4.2.4. Peritoneal Carcinomatosis

Peritoneal carcinomatosis is a form of cancer characterized by the dissemination of tumor cells on the peritoneum, the membrane that lines the abdominal cavity and its organs. It is typically a secondary cancer, most often originating from cancers of the digestive system, such as stomach, colon, rectal, and pancreatic cancers, or from gynecological cancers, primarily ovarian cancer [161]. Diagnosing peritoneal carcinomatosis is generally challenging due to its anatomical configuration. While [^18^F]FDG PET imaging is an option, false negatives can occur in cases of small tumor deposits, mucinous tumors, or gastric cancers that poorly uptake [^18^F]FDG. Additionally, it has been shown that non-malignant and inflammatory lesions can uptake [^18^F]FDG, potentially leading to false positives [162,163]. In a retrospective study of 57 patients with peritoneal carcinomatosis, Guzel’s team compared [^68^Ga]Ga-FAPI-04 with [^18^F]FDG and found that [^68^Ga]Ga-FAPI-04 images were generally characterized by low non-specific uptake in the peritoneal cavity and a high tumor-to-background ratio (14.9 vs. 6.8 for [^18^F]FDG), offering superior sensitivity compared to [^18^F]FDG PET for diagnosing peritoneal carcinomatosis (97–100% vs. 53–71%, respectively) [164]. These findings were supported by Elboga’s team, who conducted a retrospective study on 37 patients with colorectal, stomach, or pancreatic cancer. [^68^Ga]Ga-FAPI-04 PET imaging revealed a greater number of lesions in all categorized regions, with an average SUVmax of 10.7 for detecting peritoneal metastases, compared to an average SUVmax of 3.1 for [^18^F]FDG [165].

#### 4.2.5. Other Applications in Oncology

The aforementioned cancers are among those with the lowest [^18^F]FDG uptake, where FAPI imaging appears to offer the most significant benefit. However, FAPI compounds have also been studied in other tumor types, where [^18^F]FDG uptake can be variable. For instance, in a prospective study involving 13 patients with gastric adenocarcinoma, [^68^Ga]Ga-FAPI-04 demonstrated higher sensitivity (100% of lesions detected) compared to [^18^F]FDG (50% of lesions detected) in diagnosing primary lesions [166,167]. Similarly, while [^18^F]FDG is generally advantageous in diagnosing pancreatic ductal adenocarcinoma, false positives can occur in inflammatory conditions [168]. FAPI PET combined with MRI has shown superiority over FDG PET alone in 33 patients with pancreatic ductal adenocarcinoma, detecting 33.3% more metastatic lymph nodes [169]. Also, [^68^Ga]Ga-FAPI-04 and FAPI-46 have shown promise in imaging breast and ovarian cancers, providing additional or complementary information to [^18^F]FDG for detecting primary lesions or in post-chemotherapy follow-up (100% sensitivity and 95.8% specificity for FAPI-04 vs. 78.2% sensitivity and 100% specificity for FDG in the study by Kömek et al.) [170,171,172]. In contrast, [^68^Ga]Ga-FAPI-04 is not superior to [^18^F]FDG in detecting multiple myeloma, as shown in a retrospective study of 14 patients with this cancer [167]. For tumors with high [^18^F]FDG uptake, such as brain or head and neck tumors, studies do not report any particular advantage of FAPI imaging over [^18^F]FDG [173,174,175].

#### 4.2.6. FAPI Imaging Limitations

While FAPI-04 and FAPI-46 can be used in a number of non-oncological applications, their non-specific uptake in activated fibroblasts can complicate PET scan interpretations, particularly in cases of degenerative lesions and wound healing [176]. Similarly, FAPI compounds show uptake in healthy uterine tissues [177]. Another limitation is that lymph node staging may be less accurate than primary tumor detection. This can be attributed, particularly in breast cancer, to the presence of two subpopulations of CAFs within the lymph nodes: CAF-S1, which overexpresses FAP, and CAF-S4, which does not express FAP [178].

### 4.3. Other Quinoline-Based FAP Inhibitors for Diagnostic Applications Studied in Humans

Following the success of the initial FAPI series discussed above, other novel quinoline candidates have subsequently been developed.

#### 4.3.1. OncoFAP

**Figure 15:**
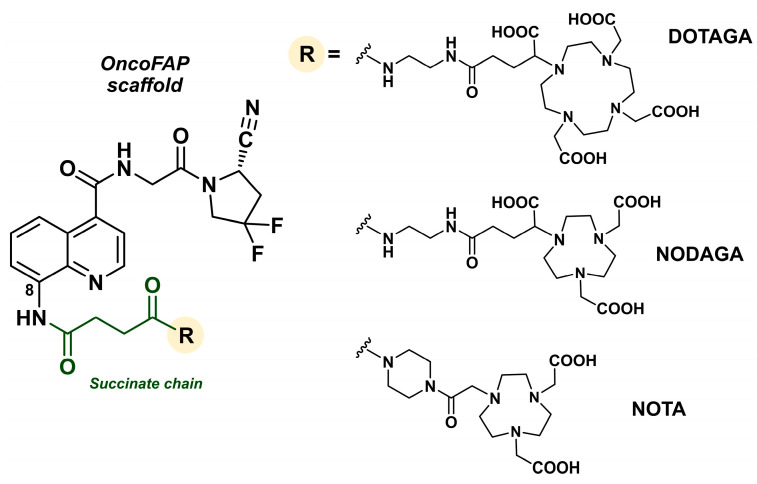
General chemical structures of OncoFAP derivatives.

Functionalization of the quinoline group at position 8 with a short succinate linker led to the discovery of OncoFAP. This FAP ligand contains a difluorocyanoproline, an 8-aminoquinoline moiety, and a carboxylic acid at the end of the succinate chain, which can be used as a conjugation motif. As a result, it can be functionalized with different chelators for radiolabeling, as well as fluorophores and cytotoxic drugs (Figure 15) [138].

In biodistribution studies in mice bearing HT-1080 FAP-expressing tumors, comparison with [^68^Ga]Ga-FAPI-46 showed significantly a higher tumor uptake and tissue-to-blood ratio for [^68^Ga]Ga-OncoFAP at 1 h p.i., with the parameters at 3 h p.i. becoming similar between the two tracers. OncoFAP was subsequently evaluated in 12 patients, 8 of whom had primary tumors, including breast cancer, fibrosarcoma, colon cancer, hepatocellular carcinoma, and pancreatic tumors. In these patients, the tracer bound to primary tumors, lymph nodes, and distant metastases while being rapidly cleared from healthy organs. Compared to [^68^Ga]Ga-FAPI-46, [^68^Ga]Ga-OncoFAP showed lower liver uptake [179].

In therapeutic applications, OncoFAP was labeled with ^177^Lu using a DOTAGA chelator and tested in athymic Balb/c AnNRj-Foxn1 mice with SK-RC52.hFAP renal carcinoma xenografts, showing a biodistribution profile favorable for therapy [179]. One study also used OncoFAP as a precursor for automated radiolabeling with various radioactive isotopes [180]. DOTAGA-OncoFAP, NODAGA-OncoFAP, and NOTA-OncoFAP derivatives were synthesized from OncoFAP-COOH and radiolabeled with ^68^Ga, ^18^F, and ^177^Lu, respectively. The radioconjugates [^68^Ga]Ga-OncoFAP and [^18^F]AlF-OncoFAP exhibited high stability (>99% intact compound) in a saline solution (0.9%) and human plasma at 37 °C for 2 h. However, stability assays of [^177^Lu]Lu-DOTAGA-OncoFAP showed radiolysis-induced degradation of approximately 10% every 24 h. This degradation was significantly reduced to around 2% over 8 days with the addition of 20 mg of gentisic acid.

The FAP-binding capability of these radiopharmaceuticals was tested on SK-RC-52.hFAP cells and wild-type FAP-negative SK-RC-52 cells. A notable difference in absolute binding values was observed for FAP-positive cells: 20% for [^177^Lu]Lu-DOTAGA-OncoFAP, 2.3% for [^68^Ga]Ga-DOTAGA-OncoFAP, and only 0.25% for [^18^F]AlF-NOTA-OncoFAP. Tests on wild-type SK-RC-52 cells showed very low non-specific binding for all compounds, indicating good specificity of the radiopharmaceuticals for FAP-expressing cells [180].

Currently, [^68^Ga]Ga-OncoFAP, developed by Philogen and Blue Earth, is undergoing a first phase I clinical trial in patients with advanced solid tumors (NCT05784597), while [^177^Lu]Lu-OncoFAP is also being investigated in a phase I clinical trial announced in late 2023.

#### 4.3.2. Aluminum [^18^F]fluoride and FAPI-42

Gallium-68 emits a β+ particle with a longer mean free path compared to fluorine-18, resulting in a lower spatial resolution than ^18^F [181]. The short half-life of ^68^Ga (68 min) necessitates meticulous planning for the preparation of the radiopharmaceutical, administration, and image acquisition, thus limiting flexibility in the imaging procedures. Chemically, ^68^Ga easily complexes with cyclic chelators such as DOTA, NOTA, or NODAGA, enabling the development of radiotracers directed to a wide range of molecular targets, giving it broad applications in molecular imaging.

**Figure 16:**
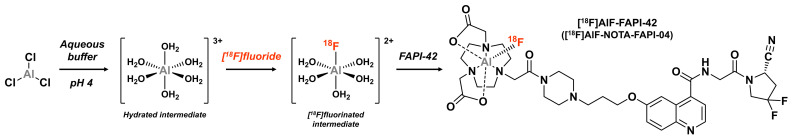
Principle of aluminum [^18^F]fluoride radiolabeling (inspired by Carroll et al. [187]), and the chemical structure of [^18^F]AlF-FAPI-42.

In contrast, ^18^F radiolabeling requires the formation of a covalent bond between the radioelement and its vector molecule. Such a bond is harder to form than the coordination bonds used for ^68^Ga complexation, requiring larger amounts of the vector (often chemically modified to increase its reactivity), harsher reaction conditions, and additional synthesis steps [182]. However, a recent non-covalent ^18^F labeling method using the strong interaction between fluoride ([^18^F]F^−^) and aluminum (Al^3+^) has overcome the chemical incompatibility between conventional radiofluorination and the cyclic chelators used for gallium. As a result, [^18^F]AlF-FAPI-42, also known as [^18^F]AlF-NOTA-FAPI-04 (Figure 16), can be produced in large quantities and benefits from a longer half-life than ^68^Ga (110 min vs. 68 min), which is advantageous in clinical settings for performing a higher number of examinations [183,184]. From a pharmaceutical standpoint, it is noteworthy that the preparation of [^18^F]fluoride aluminum-labeled FAPIs can be automated using the same synthesizers employed for the production of [^18^F]FDG [185,186].

The clinical translation of this imaging agent involved 10 patients with various cancer types (lung, pancreatic, colorectal, prostate, and lymphoma). PET scans using [^18^F]AlF-NOTA-FAPI demonstrated lower mean SUV values compared to [^18^F]F-FDG in most organs, particularly in the liver (1.1 ± 0.2 vs. 2.0 ± 0.9), brain (0.1 ± 0.0 vs. 5.9 ± 1.3), and bone marrow (0.9 ± 0.1 vs. 1.7 ± 0.4). However, the mean SUV in the pancreas (3.0 ± 2.0 vs. 1.4 ± 0.4), muscle (1.6 ± 0.4 vs. 0.7 ± 0.1), submandibular gland (3.5 ± 1.5 vs. 1.6 ± 0.5), and parotid gland (1.8 ± 0.7 vs. 0.9 ± 0.2) was higher for [^18^F]AlF-NOTA-FAPI compared to [^18^F]F-FDG. Furthermore, the fluorinated FAPI detected more lesions than [^18^F]F-FDG for certain patients [188].

**Figure 17:**
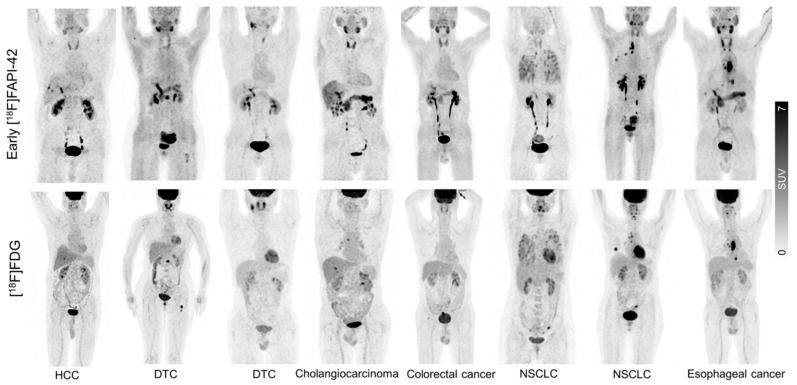
Example of maximum-intensity projection images of early [^18^F]AlF-FAPI-42 (mean = 22 min) and [^18^F]FDG PET/CT in patients with different types of cancer. HCC = hepatocellular carcinoma; DTC = differentiated thyroid carcinoma; NSCLC = non-small cell lung cancer. Images originally published by Mu et al. [191].

Similarly, a biodistribution study of [^18^F]AlF-NOTA-FAPI-04 demonstrated high specificity for FAP binding to FAP in vitro and in vivo, both on the human U87 cancer cell line (glioblastoma) and in 28 patients with various cancers (lung, pancreatic, and sarcoma). PET scans with [^18^F]AlF-NOTA-FAPI-04 gave highly contrasted images with negligible radiation exposure to healthy tissues [189]. The clinical use of this PET imaging agent has gained increasing interest, as evidenced by the numerous patient cohorts published for various oncology applications [190,191,192,193,194,195,196,197] (Figure 17).

[^18^F]AlF-NOTA-FAPI-04 also showed promise in rheumatoid arthritis; Ge et al. observed that, compared to [^18^F]FDG, [^18^F]AlF-NOTA-FAPI-04 exhibited strong uptake in inflamed joints at the early stage of arthritis, with a positive correlation between this uptake and arthritis scores [198]. Lastly, similar to FAPI analogs previously discussed, [^18^F]AlF-NOTA-FAPI-04 is currently being widely studied in the imaging of other inflammatory and fibrotic processes, as well as in cardiology [199,200,201,202].

#### 4.3.3. [^18^F]AlF-FAPI-74

FAPI-74 has a structure similar to FAPI-42 but without difluorosubstitution of the cyanopyrrolidine ring [203]. The binding properties of FAPI-74 to its molecular target were evaluated in vitro using HT-1080-FAP cells transfected with FAP, demonstrating the significant binding of [^18^F]AlF-FAPI-74. In vivo, the [^18^F]AlF-labeled tracer exhibited rapid clearance and high tumor uptake, with only trace activity found in the intestines of HT-1080-FAP xenografted mice. In the same study, [^18^F]AlF-FAPI-74 was tested on a patient with metastatic non-small cell lung cancer, showing a performance comparable to other FAPI tracers (rapid accumulation in the primary tumor and in hepatic and bone metastases, SUVmax = 6.5 ± 1.1 [1 h] and 5.6 ± 0.7 [3 h], with almost no activity in normal tissues) [182].

**Figure 18:**
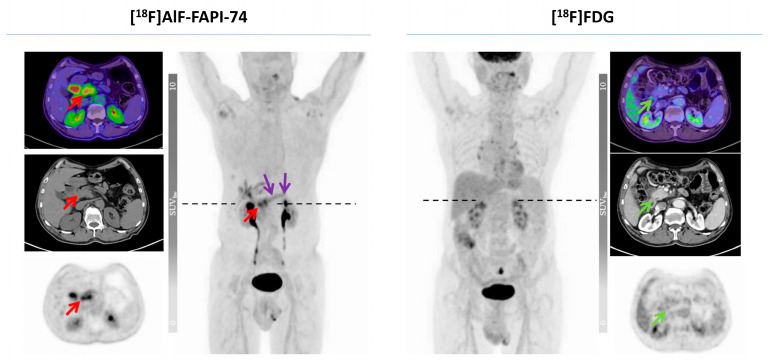
Example of additional lesion detection with [^18^F]AlF-FAPI-74 in a 69-year-old male patient who underwent preoperative assessment after neoadjuvant chemoradiotherapy to evaluate the treatment response, which revealed no notable uptake in [^18^F]FDG imaging. The red and green arrows point to the primary lesion, while the violet arrows highlight the subtle, tumor-associated pancreatitis affecting the remainder of the pancreas. The SUVmax of the primary lesion was 3.3 on [^18^F]FDG and 5.7 on [^18^F]FAPI-74, respectively. Images originally published by Novruzov et al. [204].

Giesel et al. demonstrated equal performance between [^18^F]AlF-FAPI-74, [^68^Ga]Ga-FAPI-74, and [^68^Ga]Ga-FAPI-04 in 10 patients with lung cancer (8 with adenocarcinoma and 2 with squamous cell carcinoma) [203]. However, [^18^F]AlF-FAPI-74 seems to be more frequently studied in current clinical settings, likely because gallium-based FAPI tracers, such as [^68^Ga]Ga-FAPI-74, have already been widely investigated. In a prospective study comparing [^18^F]AlF-FAPI-74 to [^18^F]FDG in seven patients with pancreatic adenocarcinoma, the biodistribution of [^18^F]AlF-FAPI-74 in normal organs was found to be comparable to ^68^Ga-labeled FAP inhibitors, except in blood, skeletal muscles, and adipose tissue, where it more closely resembled that of [^18^F]FDG. [^18^F]AlF-FAPI-74 detected 22% more lesions compared to [^18^F]FDG (32 vs. 22), including both metastatic and primary lesions (Figure 18). The SUVmax for metastatic lesions was 8.2 ± 13.9 with [^18^F]AlF-FAPI-74 and 5.7 ± 2.8 with [^18^F]FDG, while the SUVmax for primary tumor lesions was 10.5 ± 4.5 with [^18^F]AlF-FAPI-74 and 6.6 ± 3.2 with [^18^F]FDG [204].

#### 4.3.4. Technetium-99m and FAPI-34

**Figure 19:**
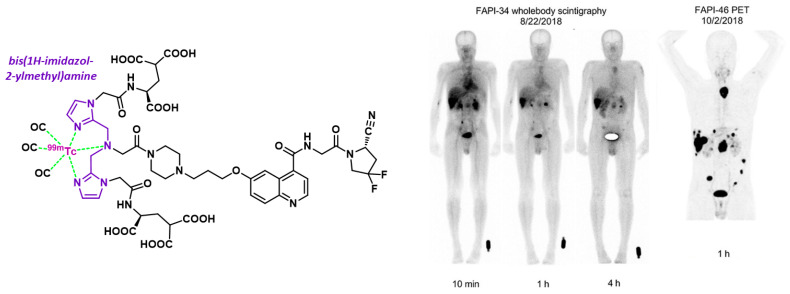
Chemical structure of [^99m^Tc]Tc-FAPI-34 and ^99m^Tc-labeled FAPI-34 planar scintigraphy images compared to ^68^Ga-labeled FAPI-46 PET imaging in a patient with pancreatic cancer. Images originally published by Lindner et al. [205].

Technetium-99m (^99m^Tc), the most commonly used gamma-emitting isotope in nuclear medicine, is readily available via ^99^Mo/^99m^Tc generators. Although this radiometal typically complexes quickly at room temperature with suitable chelating moieties, its reactivity differs from ^68^Ga, requiring the use of specific chelators. FAPI-34 was developed with a bis(1*H*-imidazol-2-ylmethyl)amine tridentate motif capable of chelating technetium in the form of the aquo tricarbonyl complex [^99m^Tc][Tc(CO)_3_(H_2_O)_3_]^+^ (Figure 19). This tracer represents a promising candidate for ^99m^Tc scintigraphic imaging due to its high contrast, achieved by significant tumor uptake and rapid clearance from the rest of the body [205]. Clinical use of [^99m^Tc]Tc-FAPI-34 has been reported in two patients with metastatic pancreatic and ovarian cancer who had previously undergone [^68^Ga]Ga-FAPI-46 PET imaging and [^90^Y]Y-FAPI-46 therapy. Follow-up imaging with [^99m^Tc]Tc-FAPI-34 revealed the same lesions on both SPECT and PET scans (Figure 19) [205]. Notably, FAPI-34 could also be used in therapy when labeled with rhenium-188 (^188^Re). In addition to FAPI-34, other quinoline-based scintigraphic imaging vectors have been developed, such as [^99m^Tc]Tc-L1, [^99m^Tc]Tc-HYNIC-Glc-FAPT, and [^99m^Tc]Tc-HYNIC-FAPI-04, each utilizing different technetium chelating groups [206,207,208].

### 4.4. Second-Generation FAP Inhibitors with a Non-Quinoline Structure

Although quinoline-based FAPI derivatives, particularly FAPI-46, are excellent imaging vectors and show considerable potential for therapeutic applications, their intra-tumoral retention time is not optimal for use in targeted radionuclide therapy (TRT) with ^177^Lu or ^225^Ac. Therefore, the development of new theranostic agents, particularly non-quinoline derivatives, has been encouraged to optimize this parameter.

TRT is associated with the emission of beta-minus or alpha particles produced by the radioisotope bound to the vector molecule. Alpha particles have a shorter range in tissues (<0.1 mm) than beta particles (approximately 2 mm for ^177^Lu), allowing them to selectively destroy targeted cancer cells and potentially limit the toxicity to nearby healthy tissues. In TRT, tumor cell death is closely linked to absorbed doses (energy deposited, measured in Grays, where 1 Gy = 1 J/kg), which cause DNA damage directly or indirectly, either through direct particle interaction or through the ionization and excitation of water, producing reactive oxygen species. Cell membranes and other cellular structures, like mitochondria, can also be damaged, leading to cell death. For the same absorbed dose, different types of radioactive particles have varied biological effects. Alpha particles have a higher linear energy transfer (LET, indicating energy deposited per unit length or volume) than beta-minus particles. In comparison to beta particles, alpha particles create a denser path of ionization and excitation, leading to complex types of cellular damage that are harder to repair, especially double-stranded DNA breaks. This explains their high relative biological effectiveness. Consequently, the modulation of the biological effects of a TRT strategy according to the selected radioisotope is currently being widely studied, including with vectors targeting the tumor microenvironment, as shown below.

#### 4.4.1. [^99m^Tc]Tc-iFAP

**Figure 20:**
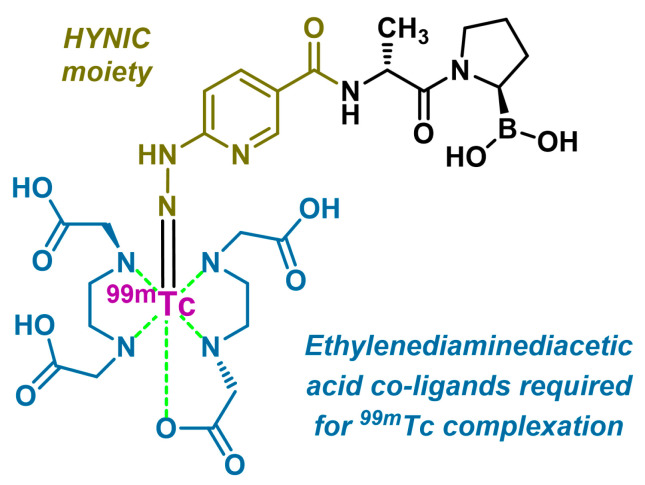
Chemical structure of [^99m^Tc]Tc-iFAP.

In 2022, Trujillo-Benítez et al. developed a FAP inhibitor based on the structure N-(pyridine-3-carbonyl)-D-Ala-boroPro, labeled with ^99m^Tc via a hydrazinonicotinic acid (HYNIC) chelator, as illustrated in Figure 20. The biokinetic profile of [^99m^Tc]Tc-iFAP is similar to that of [^68^Ga]Ga-FAPI-46 or [^99m^Tc]Tc-FAPI-34, showing rapid clearance from non-target tissues [209]. In a study involving 32 patients with either gliomas or various cancers (breast, lung, colon, NET, renal cortex, and cervical cancer), [^99m^Tc]Tc-iFAP SPECT/CT detected 100% of the primary tumors but demonstrated lower sensitivity for lymph nodes and distant metastases compared to [^18^F]FDG [210]. However, it showed good efficacy in distinguishing between high- and low-grade gliomas due to high contrast. In theranostics, the ^99m^Tc/^188^Re pair is not compatible with iFAP, because rhenium cannot complex with the HYNIC chelator, calling for the use of alternative vectors for potential therapy purposes [209].

#### 4.4.2. PNT6555

**Figure 21:**
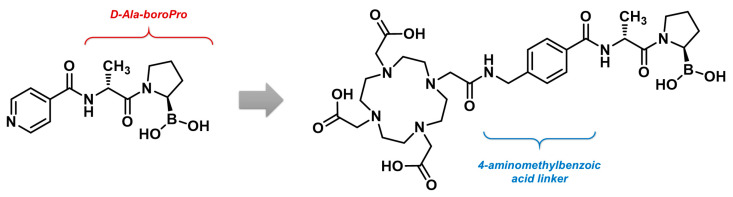
Chemical structure of initial compound 3099 (**left**) and its DOTA-containing analog PNT6555 (**right**).

A boronic acid derivative, N-(pyridine-4-carbonyl)-D-Ala-boroPro or compound 3099, was first reported by Poplawski et al. and exhibited a nanomolar affinity, along with high selectivity, for FAP compared to other enzymes in the DPP and PREP subfamilies. The combination of the D-Ala-boroPro motif with the DOTA chelator via a 4-aminomethylbenzoic acid linker led to the development of compound PNT6555, shown in Figure 21, which was studied for both diagnostic and therapeutic applications.

In murine FAP-positive tumor models, [^68^Ga]Ga-PNT6555 demonstrated selectivity for FAP-expressing tumors and reached high tumor-to-background contrast in PET imaging. Moreover, PNT6555 radiolabeled with ^177^Lu showed significant anticancer effects, outperforming [^177^Lu]Lu-FAPI-46 in terms of tumor growth inhibition after a single injection of equivalent doses. It successfully inhibited tumor growth for up to 55 days following a 30 MBq injection per mouse. By comparison, the previously mentioned study by Liu et al. showed that [^177^Lu]Lu-FAPI-46 did not produce significant tumor growth inhibition after a 30 MBq injection in mice xenografted with the same cell line [211]. However, to draw definitive conclusions, the comparison should be reproduced within the same experiment. Additionally, PNT6555 labeled with ^225^Ac demonstrated comparable activity, delaying tumor growth for 65 days at a 50 kBq dose, suggesting therapeutic versatility. The favorable preclinical results of PNT6555 support its clinical potential as an alternative to quinoline-based FAPI agents [212]. This molecule, owned by POINT Biopharma, is currently under evaluation in humans as [^177^Lu]Lu-PNT6555 in a phase I clinical trial (NCT05432193) [213]. It is being tested for various cancers, including pancreatic, esophageal, colorectal cancer, melanoma, cholangiocarcinoma, and other solid tumors with FAP overexpression [214].

#### 4.4.3. FAP-2286

**Figure 22:**
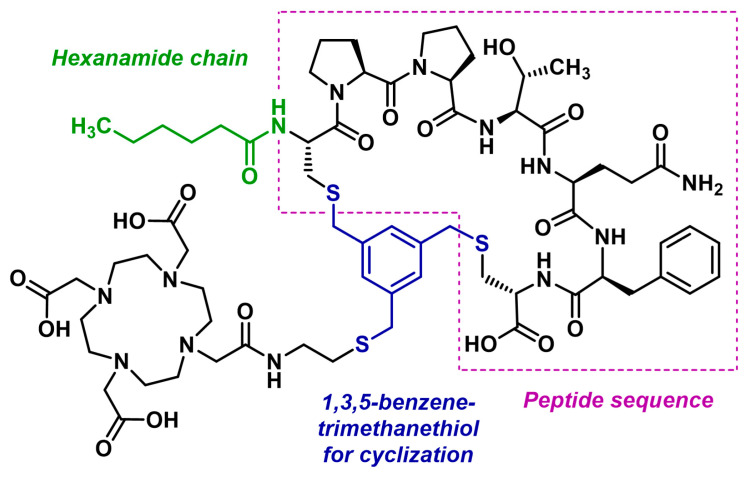
Chemical structure of FAP-2286.

The German biopharmaceutical company Clovis Oncology developed FAP-2286, a cyclic peptidomimetic composed of seven amino acids. Cyclic peptides and pseudopeptides offer the advantage of being generally more stable and rigid than their linear counterparts, often resulting in higher affinity and specificity for their targets [215]. This rigidity also gives cyclic peptides greater resistance to enzymatic degradation [216]. FAP-2286 consists of a cyclized peptide sequence linked to a DOTA chelator via a 1,3,5-benzenetrimethanethiol group (Figure 22). Complexes of FAP-2286 with ^68^Ga, ^111^In, and ^177^Lu have been studied in detail [217].

Initial studies showed that [^68^Ga]Ga-FAP-2286 exhibited a distribution profile similar to [^68^Ga]Ga-FAPI-46, with slightly higher physiological uptake in the liver, kidneys, and heart. Rapidly translated to clinical use, [^68^Ga]Ga-FAP-2286 PET imaging was performed on 64 patients, primarily with cancers of the head and neck, liver, stomach, pancreas, ovaries, and esophagus, for cancer staging or relapse identification. Among these patients, 63 also underwent a comparative PET with [^18^F]FDG and 19 with [^68^Ga]Ga-FAPI-46. The final diagnosis was based on histopathological results (58 patients) and radiological diagnosis (comprehensive imaging review; 6 patients). [^68^Ga]Ga-FAP-2286 showed lower background uptake compared to [^68^Ga]Ga-FAPI-46 in the thyroid, pancreas, muscles, and salivary glands. For tumor detection, among 44 patients requiring staging, [^68^Ga]Ga-FAP-2286 detected all primary tumors across nine different cancer types, whereas [^18^F]FDG missed nine tumors. Moreover, [^68^Ga]Ga-FAP-2286 demonstrated a higher SUVmax of 11.1 compared to 6.9 for [^18^F]FDG and a median tumor-to-background ratio of 9.2 compared to 3.0 for [^18^F]FDG, indicating superior tumor lesion detectability. [^68^Ga]Ga-FAP-2286 and [^68^Ga]Ga-FAPI-46 produced similar clinical results for tumor imaging when compared to [^18^F]FDG [218,219]. To further optimize the diagnostic properties of this vector, a NOTA analog was developed, allowing for labeling with [^18^F]aluminum fluoride [220].

Concerning the therapy counterpart, a preliminary study investigated the use of [^177^Lu]Lu-FAP-2286 in 11 patients with progressive, metastatic adenocarcinomas of the pancreas (5 patients), breast (4 patients), ovary (1 patient), and rectum (1 patient). Most patients received two treatment cycles spaced 8 weeks apart, while one patient received a single cycle, and another received three cycles. The average administered dose was 5.8 ± 2.0 GBq of [^177^Lu]Lu-FAP-2286 per cycle. [^177^Lu]Lu-FAP-2286 demonstrated prolonged tumor retention, with an effective half-life of approximately 44 h in bone metastases. With acceptable side effects, these results paved the way for larger clinical trials [221]. In this regard, the safety and efficacy of [^177^Lu]Lu-FAP-2286 are currently being evaluated in the phase 1/2 LuMIERE clinical trial, sponsored by Novartis (NCT04939610) [222]. Other smaller-scale research protocols are also actively recruiting (NCT04621435 and NCT05180162).

#### 4.4.4. 3BP-3940

**Figure 23:**
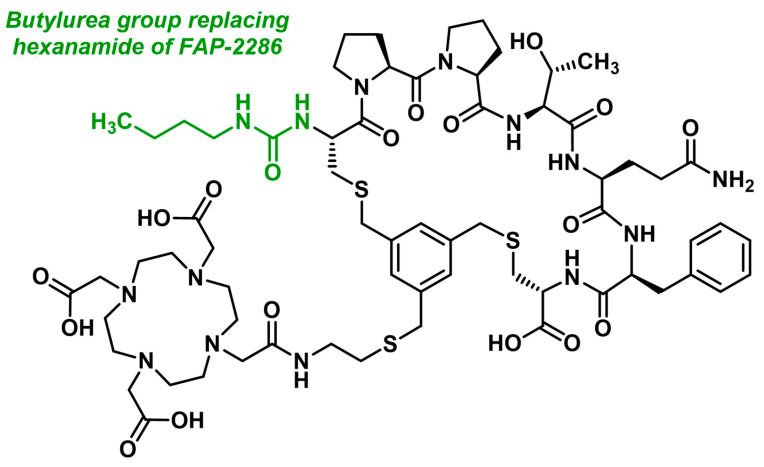
Chemical structure of 3BP-3940.

Minor structural modifications of FAP-2286 led to the development of an optimized analog, 3BP-3940 (Figure 23). Although the scientific literature on this targeting molecule is still limited, investigations have been conducted in both imaging and therapy. A patient with pancreatic cancer and liver metastases received 150 MBq of 3BP-3940 labeled with ^68^Ga for PET imaging. The scan showed intense accumulation in the primary tumor and metastatic lesions, confirming the targeting ability of 3BP-3940. Recently, another peptidomimetic with an identical sequence, designed exclusively for diagnostic purposes, was developed: [^18^F]AlF-FAP-NUR, which includes a NOTA chelator and can be radiolabeled with ^68^Ga or [^18^F]aluminum fluoride [223].

Regarding the use of 3BP-3940 in TRT, an initial patient received a single dose of 9.7 GBq of the vector radiolabeled with ^177^Lu. The treatment was well tolerated without significant changes in the vital signs or biological parameters, suggesting good tolerance of the radiocomplex [224]. Another study presents the first human results from a theranostic approach involving 3BP-3940. An initial PET scan with [^68^Ga]Ga-3BP-3940 was used to select patients for TRT with the same vector, labeled with various isotopes (^177^Lu, ^90^Y, and ^225^Ac), administered either alone or in tandem isotope combinations (^177^Lu + ^225^Ac or ^90^Y + ^225^Ac). After 1 to 5 cycles of TRT, the average cumulative activity of the different radioisotopes administered to patients was as follows: 12.6 ± 11.5 GBq for ^177^Lu (n = 21, with a maximum of 43.1 GBq); 9.8 ± 7.2 GBq for ^90^Y (n = 10, with a maximum of 25.7 GBq); and 15.2 ± 8.5 MBq for ^225^Ac (n = 23, with a maximum of 33 MBq). One patient achieved complete remission, four had partial remission, and three experienced disease stabilization. The other patients showed disease progression (n = 12). Across the cohort (n = 28), the median overall survival from the start of TRT was 9.0 months [225,226].

## 5. Conclusions

FAP targeting has emerged as a pivotal strategy for exploring the TME and enhancing cancer diagnostics. The development and current clinical use of FAPI compounds, particularly those based on quinoline derivatives, allows more precise imaging of tumors with low [^18^F]FDG uptake. To date, numerous quinoline-based FAPI derivatives are being investigated in phase 1 clinical trials, highlighting the ongoing interest in this class of radiopharmaceuticals. Of note, only three molecules have advanced to phase 2 trials: [^18^F]F-FAPI-04, [^18^F]F-FAPI-74, and [^68^Ga]Ga-FAPI-46. Of these, [^68^Ga]Ga-FAPI-46 is the most extensively studied, demonstrating potential for both diagnostic and therapeutic applications. The progression of FAPI agents into later-stage clinical trials is crucial for the broader adoption of FAP-targeted diagnostics and treatments. Additionally, the development of single-vial cold kits for efficient ^68^Ga radiolabeling of FAPI derivatives could facilitate broader clinical implementation. Furthermore, the potential industrial-scale production of covalently ^18^F-labeled FAPI derivatives offers the prospect of more widespread access to high-resolution PET imaging agents. In the future, theranostic strategies involving FAPI derivatives hold significant promise, especially through the conception and evaluation of non-quinoline compounds such as FAP-2286 and 3BP-3940, opening up avenues for personalized cancer treatment. As with other radiopharmaceuticals such as [^177^Lu]Lu-oxodotreotide in neuroendocrine tumors or [^177^Lu]Lu-vipivotide tetraxetan in metastatic prostate cancer, an important area of investigation for FAPI-based TRT will certainly be its suitability for combined strategies pairing TRT with chemotherapy, immunotherapy, or oral kinase inhibitors.

In summary, while significant progress has been made, the full diagnostic and therapeutic potential of FAP-targeting agents remains to be consolidated for potential widespread use in routine clinical practice.

## Figures and Tables

**Table 1:** Summary of the main properties of quinoline-based FAPI vector molecules. Therapeutic potential has only been indicated for vectors studied in patients.

Name	Chelator	Investigated in Clinical Setting	Radioisotope for Diagnostic	Potential for Therapy	Radioisotope for Therapy	Comments
FAPI-01	NA	No	^ 125 ^ I	No	NA	Used for biodistribution studies Susceptible to enzymatic deiodination
FAPI-02	DOTA	Yes	^68^Ga	Yes	^177^Lu	Good pharmacokinetic profile Affinity for FAP to be improved (non-fluorinated derivative)
** FAPI-04 **	** DOTA **	**Yes**	**^68^** **Ga**	**Yes**	**^177^** **Lu, ^90^Y**	**Good pharmacokinetic profile** **High affinity for FAP (fluorinated derivative)**
FAPI-06, FAPI-07	DOTA	No	^68^Ga	NA	NA	Derivatives with an amino alkyl linker Low tumor cell retention
FAPI-08, FAPI-09	DOTA	No	^68^Ga	NA	NA	Linker at position 7 instead of 6 Fast clearance from tumor cells
FAPI-10	DOTA	No	^68^Ga	NA	NA	Derivative bearing a nuclear localization signal Strong accumulation in the kidneys
FAPI-21	DOTA	Yes	^68^Ga	NA	NA	Derivative with a bridged piperazine linker Slower clearance from tumor cells Intense uptake in several non-target tissues
FAPI-34	bis- imidazolylmethyl- amine	Yes	^99m^Tc	No	NA	Derivative for SPECT imaging purposes
FAPI-36		No	^68^Ga	NA	NA	Derivative with a bridged 1,4-diazepane linker High uptake in tumor but also in non-target tissues
FAPI-39		No	^68^Ga	NA	NA	Methylene (-CH_2_-) anchoring of the linker
FAPI-40		No	^68^Ga	NA	NA	Thioether (-S-) anchoring of the linker
FAPI-41		No	^68^Ga	NA	NA	Secondary amine (-NH-) anchoring of the linker
** FAPI-42 **	** NOTA **	**Yes**	**^68^** **Ga, [^18^F]AlF**	**No**	**NA**	NOTA analog of FAPI-04
** FAPI-46 **	** DOTA **	**Yes**	**^68^** **Ga**	**Yes**	**^177^** **Lu, ^90^Y, ^225^Ac**	**Tertiary amine anchoring of the linker** **High tumor-to-healthy tissues ratio** **Best candidate in initial quinoline series**
FAPI-55	DOTA	No	^68^Ga	NA	NA	Derivative with a piperidine linker instead of piperazine High lipophilicity causing prolonged hepatic residence time
FAPI-76	NOTA	Yes	^68^Ga, [^18^F]AlF	No	NA	Non-fluorinated analog of FAPI-42

NA = not applicable. **Bold** = derivatives most widely studied in a clinical setting.

## Data Availability

No new data were created or analyzed in this study. Data sharing is not applicable to this article.
